# Heterozygous HbAC but not HbAS is associated with higher newborn birthweight among women with pregnancy-associated malaria

**DOI:** 10.1038/s41598-017-01495-9

**Published:** 2017-05-03

**Authors:** Marilou Tétard, Jacqueline Milet, Sébastien Dechavanne, Nadine Fievet, Dominique Dorin-Semblat, Jacques Elion, Rick M. Fairhurst, Philippe Deloron, Nicaise Tuikue-Ndam, Benoît Gamain

**Affiliations:** 1 Université Sorbonne Paris Cité, Université Paris Diderot, Inserm, INTS, Unité Biologie Intégrée du Globule Rouge, Laboratoire d’Excellence GR-Ex, Paris, France; 2 Institut de Recherche pour le Développement, UMR 216, Mère et Enfant face aux Infections Tropicales, Paris, France; 3 COMUE Sorbonne Paris Cité, Faculté de Pharmacie, Paris, France; 4 Laboratory of Malaria and Vector Research, National Institute of Allergy and Infectious Diseases, National Institutes of Health, Rockville, MD USA

## Abstract

Pregnancy-associated malaria (PAM) is associated with poor pregnancy outcomes. Hemoglobin S (HbS) and hemoglobin C (HbC) mutations are frequently encountered in malaria-endemic areas of Africa, where they protect children from severe and uncomplicated *Plasmodium falciparum* malaria. However, scant epidemiological data exist on the impact of these Hb variants on PAM. A prospective cohort of 635 Beninese pregnant women was recruited before 24 weeks of gestational age and followed until the end of pregnancy. HbAA, HbAC, and HbAS genotypes were determined and tested for association with pregnancy outcomes and PAM indicators using linear and logistic multivariate models. Newborns from HbAC mothers had higher birthweights than those from HbAA mothers among women infected at any time during pregnancy (mean difference 182.9 g, p = 0.08), or during the first half of pregnancy (654.3 g, p = 0.0006). No such birthweight differences were observed between newborns from HbAS and HbAA mothers. HbAC and HbAS were not associated with other pregnancy outcomes or PAM indicators. In conclusion, HbAC but not HbAS is associated with an improved birth outcome in pregnant women with documented PAM. Higher-birthweight newborns from HbAC mothers may have a survival advantage that contributes to the natural selection of HbC in malaria-endemic areas.

## Introduction

In endemic areas, the morbidity and mortality associated with *Plasmodium falciparum* malaria have naturally selected mutations in the β-chain of hemoglobin gene (*HBB*), giving rise to the HbS and HbC hemoglobinopathies^1^. While HbS is encountered at frequencies up to 18% across sub-Saharan Africa, the Middle East, and India, HbC is prevalent only in West Africa^2^. HbS homozygosity results in sickle-cell disease and is associated with high mortality rates while HbC homozygosity is clinically benign^2^, as are the heterozygous HbAS and HbAC traits. These *HBB* mutations are known to confer protection from life-threatening *P. falciparum* malaria^3–8^. For example, HbAS has been associated with 50% and 80% reduced risks of developing uncomplicated and severe malaria^6^, and HbAC and HbCC have been associated with 29% and 93% reduced risks of developing severe malaria, respectively^4^. Additional studies have confirmed the malaria-protective effects of HbC^5, 9, 10^.

Each year, over 50 million pregnant women living in malaria-endemic areas are at risk of developing pregnancy-associated malaria (PAM) caused by *P. falciparum* ^11^. PAM is characterized by massive accumulation of parasitized erythrocytes and monocytes in the placental intervillous blood spaces^12^, leading to serious adverse clinical outcomes for both the mother and the child^13, 14^. PAM-associated low birthweight is estimated to cause about 62,000 to 363,000 infant deaths every year in Africa alone^15^. Whether HbAS and HbAC confer protection against this and other detrimental PAM outcomes has not been adequately assessed. One study found no difference in the prevalence of peripheral *P. falciparum* parasitemia between pregnant HbAA and HbAS women in Gabon^16^, while another did not associate HbAS with protection from PAM or its adverse effects in pregnant women in Malawi^17^. Studying the patterns of differential susceptibility to PAM could help in identifying correlates of both malaria pathogenesis and immunity, and then candidate protective molecular mechanisms. Such mechanisms could then be the foundation for the rational design of vaccine candidates as well as preventive measures and treatments against PAM. Our study thus aimed to investigate, for the first time within a single cohort, the effects of maternal HbAS and HbAC genotypes on pregnancy outcomes.

## Results

### Study Population and *HBB* genotyping

**Figure 1:**
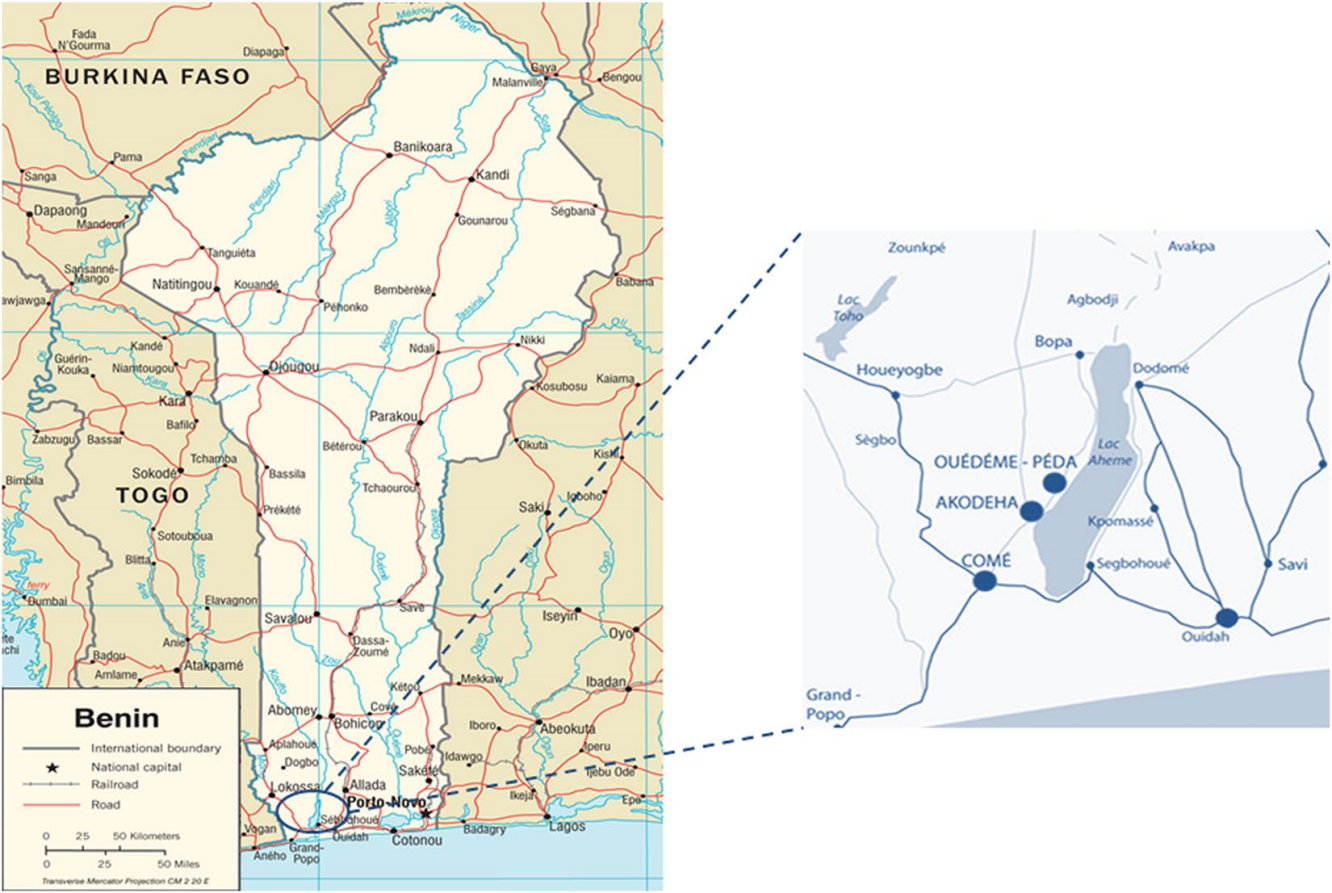
Map of the study area. The study was conducted in Comè District, 70 km west of Cotonou, Benin, where perennial malaria transmission peaks from April to July and September to November. Three dispensaries (Comè, Akodeha, and Ouedeme Pedah), 10 km away from each other, were involved. Comè is a semi-rural area and the two other sites are located in more rural settings. Maps credit: http://www.carte-monde.org/cartes-du-benin/ and P. Deloron.

The study was conducted in Comè District, 70 km west of Cotonou, Benin (Fig. 1). The characteristics of 602 pregnant women included in this study are shown in Table 1. Among them, 53 (8.8%) and 95 (15.8%) carried HbAC and HbAS genotypes, respectively. The average ages of HbAA, HbAC, and HbAS women were 27.1, 28.1, and 26.6 years, and did not differ by *HBB* genotype. Peda was the more prevalent ethnicity in HbAA and HbAS women, while Watchi was the most prevalent in HbAC women. The mean gestational age determined by ultrasound was 39.7 weeks for all three groups of women, which also did not differ in BMI, ABO blood group, or number of *P. falciparum* infections.

**Table 1:** Characteristics of pregnant women, according to *HBB* genotype.

	HbAA (n = 454)	HbAS (n = 95)	HbAC (n = 53)	*P* ^a^
**Age (years):** mean (SD)	27.1 (6.27)	26.6 (5.94)	28.1 (6.94)	0.38
**Ethnic group:** n (%)				
Peda	151 (33.3%)	29 (30.5%)	8 (15.1%)	0.06
Watchi	73 (16.1%)	18 (18.8%)	19 (35.8%)	
Adja	66 (14.5%)	13 (13.7%)	6 (11.3%)	
Saha	71 (15.6%)	16 (16.8%)	6 (11.3%)	
Mina	40 (8.8%)	7 (7.4%)	6 (11.3%)	
Other	53 (11.7%)	12 (12.6%)	8 (15.1%)	
**Primigravidae:** n (%)	79 (17.4%)	16 (16.8%)	4 (7.5%)	0.18
**Gestational age at delivery (weeks):** mean (SD)	39.7 (2.0)	39.7 (2.2)	39.8 (1.7)	0.81
**Low body mass index (**<**18.5):** n (%)	37 (8.2%)	8 (8.4%)	6 (11.3%)	0.74
**Birthweight of newborns (g):** mean (SD)^b^	3049.4 (412)	3106.1 (408)	3165.2 (453)	0.13
**Blood group:** ^c^ n (%)				
A	96 (23.8%)	23 (27.7%)	10 (21.3%)	0.26
B	100 (24.8%)	14 (16.9%)	7 (14.9%)	
O	197 (48.9%)	45 (54.2%)	29 (61.7%)	
AB	10 (2.5%)	1 (1.2%)	1 (2.1%)	
**Placental malaria:** ^d^ n (%)	35 (10.6%)	8 (11.11%)	4 (10.8%)	0.99
**Peripheral parasitemia at delivery:** n (%)	46 (10.1%)	10 (10.5%)	4 (7.5%)	0.82
**Peripheral parasitemia at recruitment:** n (%)	48 (10.6%)	10 (10.5%)	4 (7.5%)	0.82
**Number of parasite infections:** n (%)				
0	274 (60.4%)	59 (62.1%)	34 (64.2%)	0.32
1	99 (21.8%)	23 (24.2%)	15 (28.3%)	
2	54 (11.9%)	9 (9.5%)	3 (5.7%)	
>2	27 (5.9%)	4 (4.2%)	1 (1.9%)	
**Number of antenatal visits:** mean (SD)	5.5 (1.8)	4.7 (1.8)	4.4 (1.8)	0.08

^a^ *P* value of One Way Analysis of Variance for quantitative variables, and *P* value of Chi-square test for qualitative variables. In order for the Chi-square test to be considered valid, AB and B blood groups were combined, and 2 and >2 numbers of parasite infections were combined.

^b^Birthweights for 551 women with no premature births or stillbirths are excluded (417 HbAA, 88 HbAS,46 HbAC).

^c^ABO blood group data were available for 533 women (403 HbAA, 83 HbAS, 47 HbAC).

^d^Placental malaria data were available for 438 women (329 HbAA, 72 HbAS, 37 HbAC).

### Assessment of maternal *HBB* genotype on newborn birthweight

**Figure 2:**
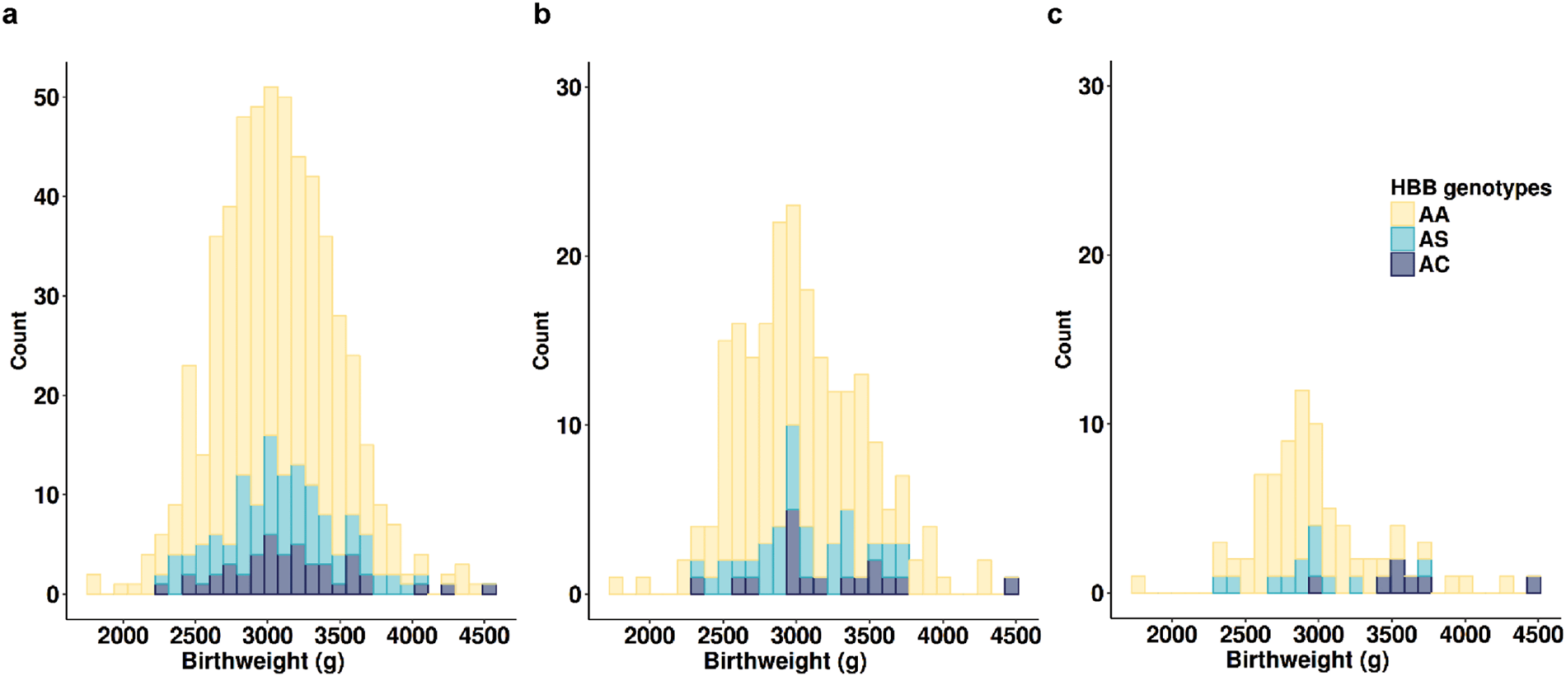
Stacked histograms of newborn birthweights according to maternal *HBB* genotypes in the whole cohort and in women with malaria infection during pregnancy. (**a**) Entire cohort (n = 551; 417 HbAA, 46 HbAC, 88 HbAS). (**b**) Mothers infected at least once during pregnancy (n = 218; 168 HbAA, 17 AC, 33 AS). (**c**) Mothers infected before 20 weeks of pregnancy (n = 80; 61 HbAA, 7 HbAC, 12 HbAS). Premature births and stillbirths were excluded.

We first assessed the effects of maternal *HBB* genotype on newborn birthweight. Analyses excluded premature births (n = 35) and stillbirths (n = 16). In the whole cohort, mean (SD) birthweights for HbAA, HbAC, and HbAS mothers were 3049.4 g (412), 3165.2 g (453), and 3106.1 g (408), respectively (Fig. 2a). In a multivariate regression model adjusted on low maternal BMI, gestational age, parity status, fetal sex, malaria infection at inclusion, ethnicity, and season at the beginning of pregnancy, no effects of *HBB* genotype in mothers were observed. Birthweights were significantly lower among primigravid mothers (mean difference −107.9 g; 95% confidence interval [95% CI] −198.8, −17.02; p = 0.02), women with parasite infection at inclusion (mean difference −137.6 g; 95% CI −258.4, −16.8; p = 0.02), and women exposed to the peak malaria season during the first trimester of pregnancy (mean difference −89.03 g; 95% CI −158.4, −19.6; p = 0.02). Mean birthweight appeared to be lower for women from Adja and Peda ethnic groups compared to the others, but no significant differences were observed between ethnic groups (global p = 0.36).

Analyses were further conducted on the sub-samples of women infected at least once during pregnancy (n = 218) and women with an early infection during pregnancy (n = 80), since we previously reported in the same cohort that only early infections during pregnancy were associated with a mean birthweight decrease of 98.5 g (95% CI −188.5, −8.5)^18^. Among women with at least one *P. falciparum* infection during pregnancy, 168, 17, and 33 of them carried the HbAA, HbAC, and HbAS genotypes, respectively. Mean (SD) birthweights in these three groups were 3013.4 g (426), 3197.7 g (507), and 3044.2 g (368) (Fig. 2b). After adjustment, although birthweights were similar among infants born to HbAA and HbAS mothers, infants born to HbAC mothers showed a trend towards higher birthweights, although this did not reach statistical significance (mean difference 182.9 g; 95% CI −18.9, 384.8; p = 0.08) (Table 2).

**Table 2:** Effect of *HBB* genotype on newborn birthweight in women infected during pregnancy.

	n	Mean birthweight difference (g)	95% confidence interval	*P*
Women infected during pregnancy^a^				
HbAA	168	Reference		
HbAC	17	182.9	−18.9, 384.8	0.08
HbAS	33	19.5	−129.9, 168.9	0.80
Women infected before 20 weeks of pregnancy^b^				
HbAA	61	Reference		
HbAC	12	654.3	298.3, 1019.1	6.51 × 10^−4^
HbAS	7	−113.5	−441.8, 214.9	0.49

Estimates of mean birthweight were obtained using a multiple linear model.

^a^Model was adjusted on gestational age at delivery, parity, fetal sex, season at the beginning of pregnancy, ethnic group, and malaria infection at inclusion. Covariates retained in the model were those associated with outcome at p < 0.20.

^b^Model was adjusted on gestational age at delivery, parity, fetal sex, season at the beginning of pregnancy, and ethnic group. All covariates were associated with outcome at p < 0.20, except parity. Ethnicity was forced in the model as it is a main determinant of birthweight.

Exposure to the peak malaria season during the first trimester of pregnancy (p = 0.0096) and parasite infection at inclusion (p = 0.05) were also associated with a lower mean birthweight.

For women infected before 20 weeks of gestation, newborn mean (SD) birthweights were 2934.1 g (416), 2918.3 g (359) and 3611.4 g (451) for mothers carrying HbAA (n = 61), HbAS (n = 12), and HbAC (n = 7) genotypes, respectively (Fig. 2c). In a multivariate regression model, HbAC mothers had significantly higher newborn birthweights than HbAA mothers (mean difference 654.3 g; 95% CI 298.3, 1019.1; p = 0.0006) (Table 2). After removing one outlier (a 4500 g newborn from an HbAC mother), this birthweight difference remained significant (418.7 g; 54.5, 783.0; p = 0.025). As in the group of infected women, newborn birthweights among HbAS and HbAA mothers did not differ (Table 2).

Since we previously reported in this same cohort that sub-microscopic *P. falciparum* infections (those detected only by PCR, not by thick blood smear examination) are associated with decreased maternal Hb levels and low newborn birthweight^19^, we tested the interactions between maternal *HBB* genotype and presence of sub-microscopic parasitemia on birthweight. Newborn birthweights among 44 mothers with sub-microscopic parasitemia early in pregnancy did not differ from those of uninfected mothers (interaction p = 0.79).

When analysing low birthweight (<2500 g) using a multivariate logistic regression model, we detected no effect of *HBB* genotype or any covariates (data not shown). However, this analysis is underpowered since low birthweights were observed in only 5.4% of newborns (after excluding premature births), and included only one from an HbAC mother and five from HbAS mothers.

### Assessment of maternal *HBB* genotype on prematurity, number of infections during pregnancy, and parasite density

We further analysed prematurity, number of infections during pregnancy, and parasite density according to *HBB* genotype. Of the 35 premature births, five and six occurred in HbAC and HbAS mothers, respectively. Premature births were associated with low BMI (p = 0.004) and first pregnancy (p = 0.05), but not *HBB* genotype (p = 0.24 for HbAC, p = 0.64 for HbAS) or *P. falciparum* infection.

**Figure 3:**
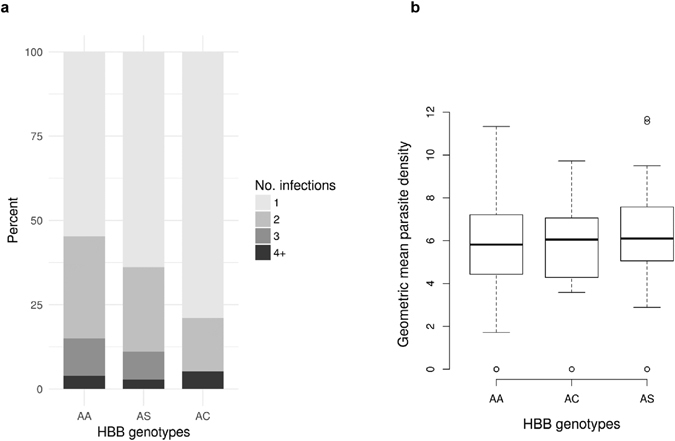
Number (**a**) and density (**b**) of *Plasmodium falciparum* infections among women infected at least once (n = 234), according to *HBB* genotype. (**a**) Stacked bar chart of the number of *P. falciparum* infections by genotype group. The number of infections per woman is divided in four categories: 1, 2, 3, or 4 and more infections during the pregnancy (grey scale). Each bar represents the percentage of each infection categories according to *HBB* genotype group. (**b**) Parasite density measured by microscopy according to genotype group. Values are log-transformed parasite densities (parasites/µl) for women with one infection or the geometric mean of parasite densities for women with more than one infection. Median, interquartile range, range, and outliers are represented.

We next investigated the relationship between *HBB* genotype and number of infections during pregnancy and parasite density (Fig. 3). HbAC women had fewer infections than HbAA and HbAS women (Fig. 3a), but this difference was not significant (Kruskal-Wallis test, p = 0.10). We also performed a multivariate analysis on the entire cohort to test for effects on the number of infections during pregnancy, after excluding women with <10 weeks of follow-up (n = 598). After adjustment on length of follow-up and season of pregnancy, increased number of infections during pregnancy was associated with low BMI (p = 0.038) and first pregnancy (p = 0.011), but not maternal HbAC (p = 0.32) or HbAS (p = 0.64) genotypes. In the subset of infected women, mean parasite densities were similar among HbAA, HbAS, and HbAC women (Kruskal-Wallis test, p = 0.73) (Fig. 3b). Therefore, the higher birthweights among HbAC mothers do not seem attributable to differences in the number or density of parasite infections.

## Discussion

Although it is well documented that HbAS confers malaria protection to African children^3, 5, 6^, HbAS had no discernible impact on newborn birthweight or other pregnancy outcomes in our study of Beninese women. These results are consistent with a recent study of pregnant Malawian women, which found no association between HbAS and PAM indicators^17^. The increased newborn birthweights we observed among HbAC but not HbAS women infected with *P. falciparum* during pregnancy suggest that these two *HBB* genotypes differentially impact PAM. Indeed, mean birthweight among HbAA and HbAS mothers, but not HbAC mothers, decreases successively in the whole cohort, the subset of all infected women, and the subset of early-infected women in our study. Early infections during pregnancy are known to impact fetal development and thus associated with the occurrence of low birthweight. Strikingly, HbAC women infected before 20 weeks of gestation delivered newborns with a much higher birthweight than did early-infected HbAA and HbAS women, and even higher than newborns delivered by uninfected women. Given our study design and the follow-up strategy, it is still possible that we missed some infections, especially those during the first trimester before the inclusion of women. This could explain the difference observed even among women classified here as uninfected, and further suggests that the protective effect of HbAC reported in this study is significant and worthy of additional investigation.

Although few HbAC primigravidae were sampled in our study, and none were infected early in pregnancy, these limitations do not account for higher birthweights among HbAC mothers because this effect was adjusted on parity in multivariate analysis. Although we observed no differences in the number or density of peripheral blood parasitemias, these data may not adequately reflect the placental parasite load and thus the severity of PAM.

It was previously shown that parasitized HbAS and HbAC erythrocytes have reduced capacities to cytoadhere to host receptors on microvascular endothelial cells, due to significantly decreased and aberrant exposure of the parasite’s cytoadherence ligand PfEMP1 on their surface^20, 21^. Since the PfEMP1 variant VAR2CSA mediates cytoadhesion to chondroitin sulfate A (CSA) on placental syncytiotrophoblast cells^22–25^, we expected to observe increased newborn birthweights among both HbAS and HbAC women resulting from similarly reduced parasite sequestration in the placenta.

It is therefore possible that HbAC increases birthweights among early-infected women by a different mechanism, or that HbAC more strongly impairs parasite cytoadherence in the placenta, where blood flow in the intervillous space is slower than in the microvasculature, and where CSA (instead of CD36) is the predominant host cytoadherence receptor. In support of this latter hypothesis, two recently-published studies have shown that in static adhesion assays, VAR2CSA-expressing FCR3 parasites in HbAS and HbAC erythrocytes exhibit significant reductions in adherence to CSA compared to those grown in HbAA erythrocytes, this decreased binding being much greater for parasitized HbAC than HbAS erythrocytes^26, 27^. However, these studies assessed only a single parasite strain in static but not flow adhesion assays. This observation supports and could explain our finding of a protective effect of HbAC, but not HbAS, against PAM.

This study was primarily designed to characterize the effects of malaria infections in pregnant women, rather than the effects of hemoglobin variants, and thus has limitations. The main limitation is obviously owing to the size of the study population. Although a significant number of women were included, only limited proportions of them carried HbS (15.8%) or the HbC (8.8%) genotypes, or were infected (36.2%) during pregnancy. Therefore, selected analyses were conducted on a limited number of women, and were relatively underpowered. Nevertheless, this does not diminish the value of our main significant finding that infected HbAC mothers had significantly higher newborn birthweights than infected HbAA mothers. Secondly, for obvious ethical reasons, all women received two doses of sulfadoxine-pyrimethamine as intermittent preventive treatment during pregnancy, and were also treated if an infection was detected at unscheduled visits or when they presented to clinic for health-related reasons. Therefore, our results are likely to be hampered by the effects of these drug treatments.

In conclusion, we report for the first time that HbAC is associated with an improved birth outcome in infected pregnant women, which may perhaps contribute to the natural selection of HbC in the human populations of malaria-endemic West Africa. Further studies are needed to confirm that HbAC is associated with an improve birth outcome in women with PAM, and to discover the precise mechanism by which HbAC increases newborn birthweight in order to recapitulate it as a new therapy or vaccine against PAM.

## Methods

### Ethics statement

The study received ethical approval from the Comité Consultatif de Déontologie et d’Éthique of IRD in France and the Comité d’Éthique de la Faculté des Science de la Santé, Université d’Abomey Calavi in Benin (FSS 026/2007/CE/FSS/UAC). All study procedures were performed in accordance with the institutional policies, guidelines and regulations pertaining to research involving human subjects. All adult participants, or the adult caretakers of minor participants, provided written informed consent.

### Study area and population

The study was conducted between 2008 and 2011 in Comè District (Fig. 1), a semi-rural area 70 km west of Cotonou, Benin, where perennial malaria transmission peaks from April to July and September to November. A detailed description of the study area has been given elsewhere^18, 28, 29^. At the time of follow-up, the entomological inoculation rate was 35–60 infective bites per person per year^30^ and *P. falciparum* was the predominant species.

A prospective cohort of pregnant women was recruited before 24 weeks of gestation. Women were followed monthly from inclusion to delivery, and received two doses of sulfadoxine-pyrimethamine as intermittent preventive treatment during pregnancy (IPTp) per national guidelines. At inclusion, at each antenatal visit, and at unscheduled visits, when women presented to the clinic for health reasons, a rapid diagnostic test (Parascreen™, Zephyr Biomedicals, Goa, India) for *P. falciparum* infection was performed on capillary blood, venous blood was drawn, and Hb concentration was measured by the Hemocue® Hb 201^+^ system (HemoCue AB, Angelholm, Sweden).

Pregnancy was followed by four transabdominal ultrasound scans with a portable system (Titan, Sonosite, Bothell, WA) by specifically trained midwives. The first scan estimated the gestational age, and the others assessed intrauterine growth and fetal morphology. Placental and peripheral blood samples were collected at delivery. Thick blood smears were prepared from all peripheral blood samples, and Giemsa-stained *P. falciparum* parasites were counted against 200 leukocytes by two independent experienced microscopists, achieving a detection threshold of 40 parasites/µl. Four 50 µl drops of blood were spotted onto Whatman 3 paper, dried at room temperature, and stored until DNA was extracted by the Chelex method^31^. A real-time PCR assay to detect sub-microscopic *P. falciparum* infection was performed on samples collected at the times of inclusion, second IPTp uptake, one month before delivery, and at delivery^19^.

### *HBB* genotyping

*HBB* genotyping was performed on the first exon of the β-globin gene amplified (678 bp) by polymerase chain reaction (PCR) with Jump Taq (Sigma). The following oligonucleotides were used in the PCR reaction: oligonucleotide sense 5′-AGGAGCAGGGAGGGCAGGAG-3′ and antisense 5′-GCAATCATTCGTCTGTTTCCCA-3′. PCR products were sent to GATC Biotech (Mulhouse, France) for Sanger sequencing.

### Statistical analysis

Linear and logistic multivariate models were used to test the effects of HbAC and HbAS on pregnancy outcomes (newborn birthweight, low birthweight (<2500 g), premature birth, maternal anemia at delivery) and PAM indicators (number and density of *P. falciparum* infections during pregnancy). Data from 33 women were excluded from analysis due to the presence of HbCC (n = 2) or HbSC (n = 3), lack of prenatal consultation (n = 10), or twin pregnancy (n = 18). Covariates for adjustment in multivariate models were selected on the basis of previous works published by our team on birthweight and maternal anemia in the same cohort^18, 19^. We considered gestational age at delivery (estimated by an ultrasound scan at inclusion, and transformed into a four-class variable using quartiles); low body mass index (BMI) at inclusion (<18.5, ≥18.5); gravidity (primigravid, multigravid); fetal sex; and the number of antenatal visits; *P. falciparum* infection status during pregnancy (0, ≥1); total number of *P. falciparum* infections during pregnancy (0, 1–2, ≥3); *P. falciparum* infection at inclusion (yes, no); and exposure to the peak malaria season during the first trimester of pregnancy (beginning of pregnancy between April and September). Then a stepwise procedure with a backward elimination was used to choose the covariates to retain in the final models (p < 0.20). For birthweight outcome, as malaria infection may act as a modifier of the relationship between *HBB* genotype and birthweight, analyses were performed on all women; a subset of women infected at least once during pregnancy; and a subset of women infected in the first 4 months of pregnancy.

During the first 4 months of pregnancy, the effects of sub-microscopic parasitemia (detected by PCR, not thick blood smear examination) were also assessed. To distinguish the proper effect of sub-microscopic parasitemia, a three-class variable was defined: “microscopically infected” (at least one infection detected by thick blood smear), “sub-microscopically infected” (one infection detected by PCR but not by thick blood smear), and “not infected” (otherwise). This variable was included in the linear multivariate model of birthweight on the whole cohort. The interaction between infection status during the first 4 months of pregnancy and *HBB* genotype was also tested.

The number of infections detected by microscopy during pregnancy was compared using a negative binomial regression on the entire cohort and on a subset of women infected at least once. Analyses were performed after excluding women with <10 weeks of follow-up and were adjusted on the length of follow-up (in weeks), BMI, ABO blood group, gravidity, and season of pregnancy. Among women with at least one infection, the mean parasite densities by subject were compared within *HBB* genotype groups using Kruskal-Wallis test. For each subject, we computed the mean of log-transformed parasite densities observed by microscopy.

All regression models were adjusted on ethnic group. Analyses were performed using R software v3.2.3 (http://www.R-project.org/)^32^.
